# The Prediction of Malignancy Risk in Thyroid Nodules Classified as Bethesda System Category III (AUS/FLUS) and the Role of Ultrasound Finding for Prediction of Malignancy Risk

**DOI:** 10.7759/cureus.17924

**Published:** 2021-09-13

**Authors:** Awad S Alshahrani, Abdulrahman S Alamri, Abdulrahman H Balkhoyor, Moeber M Mahzari, Saeed S Alshieban, Pharaon M Majed

**Affiliations:** 1 Department of Adult Endocrinology, King Abdulaziz Medical City, Riyadh, SAU; 2 College of Medicine, King Saud bin Abdulaziz University for Health Sciences, Riyadh, SAU; 3 Department of Internal Medicine, King Abdulaziz Medical City, Riyadh, SAU; 4 Population Health Research, King Abdullah International Medical Research Center, Riyadh, SAU; 5 Department of Pathology and Laboratory Medicine, King Abdulaziz Medical City, Riyadh, SAU; 6 College of Applied Medical Sciences, King Saud bin Abdulaziz University for Health Sciences, Riyadh, SAU

**Keywords:** thyroid neoplasms, thyroid nodule, thyroidectomy, tertiary care center, saudi arabia

## Abstract

Objective

To predict the risk of malignancy in category III of the Bethesda System for Reporting Thyroid Cytopathology "Atypia of Undetermined Significance/Follicular Lesion of Undetermined Significance (AUS/FLUS)" at King Abdulaziz Medical City, Riyadh, Saudi Arabia. It also intends to determine other possible contributing predictors of malignancy in thyroid nodules such as age, sex, and ultrasound (US) findings.

Method

This retrospectively designed study included 187 patients (mean age, 43.9 ± 14.1 years) with thyroid nodules, which were diagnosed as AUS/FLUS and all patients included had total thyroidectomy or lobectomy between January 2013 and December 2018 at King Abdulaziz Medical City in Riyadh, Saudi Arabia. The electronic medical records, US images, and final cytopathology and histopathology reports were reviewed and analyzed.

Result

The overall incidence of AUS/FLUS was (46.5%). Multivariate analysis of US features revealed that malignancy was significantly associated with nodules with irregular margins, microcalcification, multiple numbers (P < 0.001), and hypoechogenicity (P 0.04).

Conclusion

Despite the high rate of malignancy of nodules AUS/FLUS, it is still consistent with previously reported studies. The highly suspicious ultrasound features (irregular margins, microcalcification, multiple nodules, and hypoechogenicity) could be helpful in the diagnosis of thyroid cancer.

## Introduction

Thyroid malignancy is the most common endocrine cancer, and it represents less than 1% of all human cancers. The annual incidence of thyroid cancer is considerably affected by geographic location, age, and sex [1]. In the Gulf Cooperation Council (GCC) countries, thyroid cancer is the fifth most common cancer type. A total of 5587 thyroid malignancies (5.9%) were diagnosed between 1998 and 2007. In general, the overall adjusted incidence rate (ASR) was 5.9 and 1.8 per 100,000 for females and males, respectively [2]. Thyroid malignancy has a higher incidence rate among females in all GCC countries and is the second most common malignancy among females. According to the 2008 Saudi Cancer Registry report, a total of 727 cases of thyroid cancer were diagnosed; 606 in Saudi patients and 121 in non-Saudi patients. In Saudi patients, thyroid cancer was 6.8% of all newly diagnosed cases of thyroid cancer that year. Thyroid cancer was the second most common cancer among females and thirteenth among males, affecting 131 (21.6%) males and 475 (78.4%) females [2].

The current standard of care in the diagnosis of thyroid cancer is the use of fine-needle aspiration cytology (FNAC), as it is the most accurate and cost-effective method for evaluating thyroid nodules, with cytopathology reporting based on The Bethesda System for Reporting Thyroid Cytopathology (TBSRTC) as recommended by the 2015 American Thyroid Association (ATA) management guidelines. The first edition of TBSRTC was introduced in 2009, followed by the 2018 second edition [3]. It classifies FNAC diagnoses into six different cytopathology categories; each category has implied cancer risk, which ranges from 0% to 3% for the benign category and up to 100% for the malignant category [4].

Among the categories in the Bethesda System, Atypia of Undetermined Significance/Follicular Lesion of Undetermined Significance (AUS/FLUS) has been debated. It represents cytological specimens that are not easily classified into benign or malignant categories [4-5]. In the first edition, the malignancy rate of this category has been suggested to be between 5% and 15% [4-5]. AUS/FLUS is reserved for specimens that contain cells (follicular, lymphoid) with architectural atypia or atypical cells that aren't considered suspicious for a follicular neoplasm (FN) or malignancy. Atypia, on the other hand, is a more prominent feature than benign changes [4]. In 2018, TBSRT was updated, and the risk of malignancy increased to 10-30% (including non-invasive follicular thyroid neoplasm with papillary-like nuclear features (NIFTP)) but remained relatively unchanged (6-18%) when excluding NIFTP as a diagnosis of benign thyroid condition [6].

There has been significant variability between cytopathologists, as much as institutions who use these categories [7-9]. Ultrasound features play a complementary role in the diagnosis of thyroid nodules with AUS/FLUS. Highly suspicious ultrasound features could be helpful in the diagnosis of thyroid cancer [10].

## Materials and methods

A retrospective study and chart review of the electronic medical record were performed following the approval of the Institutional Review Board at King Abdullah International Medical Research Center. Data were collected from electronic medical records at King Abdulaziz Medical City, Riyadh, Saudi Arabia, between January 2013 and December 2018.

The included cases in this study were the outpatients who presented with thyroid nodules and were diagnosed with AUS/FLUS based on the initial FNAC and underwent thyroidectomy or lobectomy with the postoperative histopathologic examination regardless of whether the FNAC was repeated or surgery was immediately performed. Those who did not undergo surgery or those patients for whom data was incomplete were excluded from the study. Patient demographics, preoperative ultrasound (US) features and follow-up data were collected and correlated with the final histopathologic diagnosis. The collected data were analyzed through Microsoft Excel (Microsoft Corporation, Redmond, WA).

Variables selection

Demographic variables, including gender and age of presentation with a thyroid nodule diagnosed with "Atypia of Undetermined Significance/Follicular Lesion of Undetermined Significant"(AUS/FLUS), were collected. Ultrasound feature variables of thyroid nodules were collected from the radiological reports written by radiologist consultants. Those included number, size, site, margin, echogenicity, microcalcification, and internal vascularity. The final histopathology variables were collected from the pathology report and then divided into benign and malignant cases (into papillary, poorly differentiated, and anaplastic).

Analysis

Categorical variables, including age, sex, and final histopathology, were summarized into frequency and percentages while the US features to compare between benign and malignant thyroid cancer were analyzed by using the chi-square test. Results were reported as proportions, percentages, and P-values. Significance was declared at alpha less than 0.05. Statistical analysis was performed using SPSS version 26 (IBM Corp., Armonk, NY).

## Results

A total of 187 patients with nodules who were diagnosed as (AUS/FLUS) were involved and confirmed with operative excision, of which 100 (53.3%) were benign and 87 (46.5%) were malignant. Of the 87 histologically confirmed malignant lesions, 80 (92%) were diagnosed as papillary carcinoma, six (6.9%) as follicular carcinoma, and one (1.1%) as poorly differentiated carcinoma (Table 1).

**Table 1:** Demographic data

Factors	Number (%)
Gender	
Male	29 (15.5%)
Female	158 (84.5%)
Age, mean	43.9 ± 14.1 years

The mean age of the patients was 43.9 ± 14.1 with no significant difference between benign and malignant (P=0.837). Twenty-nine cases (15.5%) were male; 15 of them were benign and 14 were malignant. For the 158 cases (84.5%) of the female group, 85 were benign and 73 were malignant with no significant difference (P=0.837) (Table 2).

**Table 2:** Percentage of malignant and benign histopathology

n=187		
Histopathology		
Benign	100 (53.5%)	
Malignant	87 (46.5%)	
Type of Malignancy (n=87)		
Papillary	80 (92%)	
Follicular	6 (6.9%)	
Poorly differentiated	1 (1.1%)	
Anaplastic	0 (0%)	

No statistically significant difference in regards to the size of benign and malignant nodules was seen (P=0.285). Multinodularity was more likely to be associated with a malignant diagnosis (P=<0.001). A similar trend was seen with the irregularity of the margins (P=<0.001). There is no significant difference in solid components between benign and malignant (P=0.80). Hypoechogenicity nodules were more likely to be associated with a malignant diagnosis (P=0.044), as was the presence of microcalcifications (P=<0.001). Regarding the internal vascularity of the nodules, there was no significant difference between benign and malignant nodules (Table 3).

**Table 3:** Comparison of demographic and ultrasound findings between the malignant and benign groups

Factors	Benign (n=100)	Malignant (n=87)	P value
Gender			0.837
Male	15 (15%)	14 (16.1%)	
Female	85 (85%)	73 (83.9%)	
Age (years)	43.55 ± 15.3	44.32 ± 12.7	0.947
Nodule Size			0.285
Less than 2 cm	25 (25%)	31 (35.6%)	
2-4 cm	54 (54%)	40 (46%)	
More than 4 cm	21 (21%)	16 (18.4%)	
Nodule Site			<0.001
Right	28 (28%)	14 (16.1%)	
Left	27 (27%)	7 (8%)	
Bilateral	45 (45%)	66 (75.9%)	
Nodule Number			<0.001
Single	47 (47%)	18 (20.7%)	
Multiple	53 (53%)	69 (79.3%)	
Nodule Margin			<0.001
Regular	59 (59%)	18 (20.7%)	
Irregular	41 (41%)	69 (79.3%)	
Solid Components			0.080
Less than 50%	15 (15%)	6 (6.9%)	
More than or equal to 50%	85 (85%)	81 (93.1%)	
Echogenicity			0.044
Hyperechogenicity	5 (5%)	4 (4.6%)	
Isoechogenicity	13 (13%)	2 (2.3%)	
Hypoechogenicity	28 (28%)	33 (37.9%)	
Heterogenicity	54 (54%)	48 (55.2%)	
Calcification			<0.001
No	69 (69%)	35 (40.2%)	
Yes	31 (31%)	52 (59.8%)	
Internal Vascularity			0.931
No	27 (27%)	23 (26.4%)	
Yes	73 (73%)	64 (73.6%)	

## Discussion

This study predicts the rate of malignancy and related risk factors in the surgical pathology of the Bethesda System Category III thyroid nodules at King Abdulaziz Medical City in Riyadh, Saudi Arabia. Several studies show that females have higher rates of malignancy, so it is not surprising that in this study, women have a higher risk of thyroid cancer than men [2,11]. And in this report, papillary thyroid cancer was the most common type of differentiated thyroid cancer in a patient with TBSRTC III, which is the same result as a recently published study in Saudi Arabia [12].

TBSRTC is the most accurate and cost-effective method for evaluating thyroid nodules for reporting FNA results for various thyroid lesions [3]. This study found that the overall malignancy rate of Bethesda System Category III was 46.5%, which is higher than that identified by the Bethesda system (about 10-30% or 6-18% after excluding NIFTP) [5]. NIFTP was not included in this study, as its diagnosis is difficult by cytology. The malignancy rate described in this study was also higher than the recently published study that was done in a tertiary hospital in Saudi Arabia, which was 27.6% in Bethesda System Category III [12]. This malignancy rate (46.5%) is consistent with the value reported in previous studies (ranging from 6% to 48%) [13-14]. The reason for these differences may stem from the heterogeneity of the group, the higher cancer risk in certain practice settings, subjective analysis by pathologists, and overuse of AUS and FLUS [12,15].

The higher malignancy rate is thought to be due to advanced cases at King Abdulaziz Medical City, Riyadh, Saudi Arabia, a tertiary referral hospital, and incidental findings of other nodules that were not aspirated after total thyroidectomy, and most of the patients underwent total thyroidectomy instead of lobectomy. And another reason is including the initial FNAC with Bethseda System Category regardless of the result of repeated FNAC that may show Bethseda System Category V or V1. A recent study in Saudi revealed that 10 of the 29 patients with repeated Bethesda III exhibited a rate of malignancy, which is 34.4% malignancy [12]. And another recent study shows 63.7% of Bethesda III patients with repeated cancer were found to have malignancy [11].

In the center, molecular markers are not studied, which could help in clinical management and could prevent unnecessary surgery. The use of these and other emerging molecular markers will likely improve the diagnosis of malignancy in thyroid nodules as well as facilitate more individualized operative and postoperative management. [16].

Those with the US findings of multiple nodules, irregular margins, microcalcification, and marked hypoechogenicity have a high risk of malignancy; thus, a surgical approach may be seriously considered for them. While those with the US findings of benign features and are not large, a follow-up monitoring may be considered. In other words, this study showed the rate of US features such as hypoechogenicity, non-circumscribed margins, microcalcification, and nonparallel shape were significantly higher in malignancy compared to benignity as shown in a similar previous study [10].

The suggested approach is to follow strict criteria developed by cytologists to limit the undercooling diagnosis of AUS/FLUS and using molecular markers to facilitate clinical diagnosis and management. This study demonstrated that the ultrasound findings could give more than the expected information to facilitate accurate diagnostic approaches in nodules with AUS/FLUS.

## Conclusions

Despite the high rate of malignancy of nodules AUS/FLUS, it is still consistent with the previously reported studies. The highly suspicious ultrasound features (irregular margins, microcalcification, multiple nodules, and hypoechogenicity) could be helpful in the diagnosis of thyroid cancer.
